# The Ability of Narcotic Detection Canines to Detect Illegal Synthetic Cathinones (Bath Salts)

**DOI:** 10.3389/fvets.2019.00098

**Published:** 2019-04-09

**Authors:** Vanquilla Shellman Francis, Howard K. Holness, Kenneth G. Furton

**Affiliations:** Department of Chemistry and Biochemistry, International Forensic Research Institute, Florida International University, Miami, FL, United States

**Keywords:** canines, bath salt, training, volatiles organic compounds, cathinones

## Abstract

Twelve certified narcotic detection canines were tested for their ability to detect confiscated illegal synthetic cathinones (bath salts). These canine teams were randomly assigned to two different groups and each group imprinted on one of two types of bath salts, ethylone and alpha-pyrrolidinovalerophenone (α-PVP), over the period of 1 month; while simultaneously documenting the imprinting procedure. The newly imprinted canines were validated by field testing and found to not only detect the imprinted bath salt to which they were trained, but they were able to detect other bath salts. The imprinting procedure and results are the first scientifically validated studies on the ability of canines to detect these harmful and illegal substances. Analytical headspace analysis using Solid Phase Microextraction (SPME) on several ethylone and α-PVP samples revealed compounds common in both. These compounds can be used to create a safe and reliable synthetic cathinone mimic training aid for canine teams.

## Introduction

Currently, there are thousands of canine teams within the United States deployed for the detection of narcotics, explosives, cadavers, live humans, ignitable liquids, biological threats, currency, and various forms of agricultural contraband (1–4). Deemed the “Gold Standard” for detection, canines are efficient, cost effective, fast, easy to train and are more sensitive than most instrumental detection devices (2, 5, 6). A major advantage for canine detection is the dog's ability to locate a target odor while simultaneously ignoring all interfering non-targeted odors (6–8).

Through the use of active sniffing (inhaling short voluminous breathes), as is the case during a field search, and the possession of more than 200 million olfactory cells; a canine's short breathes enhance the amount of odorous compounds that flow through the nostrils into the olfactory organs (6–8). The canine is then aware that there is an odor and begins to determine whether they recognize that odor or not. Although canines possess a smaller brain in comparison to their human partners, a canine's olfactory bulb is three times the size of a humans (8). This explains their increased olfactory sensitivity and why a canine can detect odor from a given substance while a human deems it odorless.

With the overwhelming amount of discrimination that can be achieved by using canines for detection of substances; the first step is training the dog to make an association with the particular target substance. A canine's olfactory neurons live for approximately 30–60 days before they die and are naturally replaced with new ones (7, 9). When a dog becomes routinely exposed to a certain odor for detection and is rewarded, there is a shift in the neurons produced. This means the newer neurons will contain more of the receptor sites of the odors that the canines are routinely encountering; thereby increasing precision and accuracy for detection of that substance (7, 9). The detection of the substance is called an “alert” defined as a characteristic change in ongoing behavior in response to a trained odor/scent, as interpreted by the canine handler. The components of the alert may include: change of behavior (COB), interest, and final response or indication.

In an effort to standardize training practices used by different canine organizations, working individuals gathered nationally to develop best practice guidelines for properly caring, training, and testing any canine for detection work. The Scientific Working Group on Dog and Orthogonal detector Guidelines (SWGDOG, www.swgdog.org) developed these best practice guidelines. SWGDOG was a federally funded partnership between local, state, federal, and international agencies dedicated to improving the reliability, accuracy, consistency of detector dog teams (10). The guidelines set forth by this group have been used and cited by numerous agencies; including the work conducted here. This work is now being continued through the Dogs and Sensors Subcommittee of the Organization of Scientific Area Committees (OSAC).

Traditional target odors for narcotic detection canines typically include: marijuana, heroin, cocaine, methamphetamine, and any other substance required to meet the training objectives (11, 12). The concern for canines and the method in which the drugs are introduced for training purposes has brought about the need for safe alternatives that still yield positive results. However, synthetic cathinones (bath salts) are not included as one of these substances for narcotic detection canines.

Instances of synthetic cathinone or bath salt intoxication by substances abusers has been increasing due to over usage. Oftentimes users of these substances will increase their intake for an increased feeling or longer duration of euphoria. Others will overlay doses in an effort to stop the adverse effects of coming off the drugs, during the down phase. When an individual consumes quantities outside the typical range for bath salts they experience increased psycho-stimulant effects such as paranoia, hallucinations, excessive agitation, anxiety, talkativeness, time lost, sweating, vomiting, muscle twitch, suicidal thoughts, tachycardia, vertigo, and many more (13–16). The length of time these adverse effects last can range from hours to months, with some cases resulting in death. On September 8th 2011, in an effort to combat the drastic increase of cases pertaining to bath salt overdosing; the Drug Enforcement Administration (DEA) issued a notice of intent to temporarily schedule three synthetic cathinones [mephedrone, methylone, and Methylenedioxypyrovalerone (MDPV)] under the Controlled Substance Act (CSA)(17, 18). The notice was issued as a response to the “imminent hazard to the public's safety” in regards to the listed drugs. Though the DEA's emergency schedule banned the possession and consumption of the previously listed drugs, amateur chemists continue to modify these compounds to avoid such regulations. By slightly altering the chemical structure of these drugs, new generations of these substances are created with slightly different chemical structures, thereby avoiding DEA regulation, while producing the similar euphoric effects when abused. The ability to quickly and inexpensively modify these drugs has made control of these substances particularly challenging for law enforcement, requiring them to use new tools to detect and confiscate these rapidly evolving bath salts.

Studies have shown that by performing simple google searches of names such as “bath salts” or “ivory wave,” consumers are brought to secure websites for retail or wholesale of various types (19). Websites routinely advertise bath salts as “legal highs,” where encryption is implemented for consumer safety, “buy one get one” advertised specials, expedited shipping, and many more aggressive marketing tactics are openly used to encourage sales of these narcotics (20, 21). Although regulations have been placed to halt incoming traffic of these drugs, a large portion still remains readily available throughout many local neighborhoods at gas stations and corner stores (The Schedule of Controlled Substances at 21 CFR 1308.11).

To the authors' knowledge, no certified narcotics detection canines are able to detect these bath salts, which leaves a significant gap for law enforcement to find and seize these substances. Field detection using canines offers a solution to the overwhelming problem with the increasing influx of these drugs into the United States that go undetected by standard procedures currently employed.

This study established whether canine teams currently certified for narcotic detection can alert to various types of synthetic cathinones (bath salts) and demonstrated the feasibility to imprint and train these canines to detect bath salts.

## Materials and Methods

All canines teams used for the study were previously certified following guidelines of the International Forensic Research Institute (IFRI) for narcotics detection (https://ifri.fiu.edu/research/detector-dog-research/index.html). The teams tested were trained and certified to detect a wide range of routinely encountered narcotics including cocaine, marijuana, heroin, methamphetamine, and MDMA.

All synthetic cathinones used were provided after special permission and under onsite supervision at the Miami Dade Police Department (MDPD, Doral, FL) and the Palm Beach Sheriff's Office (PBSO, West Palm Beach, FL). Confiscated samples used included α-PVP, methylone and ethylone; these were verified using headspace gas chromatography mass spectroscopy. Headspace analysis was conducted using polydimethylsiloxane/divinylbenzene (PDMS/DVB) solid phase microextraction (SPME) fibers. SPME fibers were exposed for 6 h to samples and then analyzed via gas chromatography mass spectrometric (GCMS) analysis. The GCMS used was a Varian 3800 GC and Saturn 2000 Ion Trap MS, equipped with a Solgel-wax capillary column, 30 m length, 0.25 μm phase thickness, and 0.25 mm internal diameter using helium a carrier gas at 1 ml per minute. Heroin, MDMA, methamphetamine, and marijuana samples were provided by the canine teams deployed by the Miami Dade Police Department and Palm Beach Sheriff's Office. Canine trials were performed on location at the Miami Dade Police Department crime lab (Doral, FL). Additional testing was conducted at Palm Beach Central High School (West Palm Beach, FL) using hallway lockers. The imprinting process was performed at the Palm Beach Sheriff's Office Canine Training Facility (Palm Beach, FL). The containers used during the imprinting phase were K-9 BSD-2 HDPE Kit purchased from EliteK9 (Boaz, KY).

**Figure 1:**
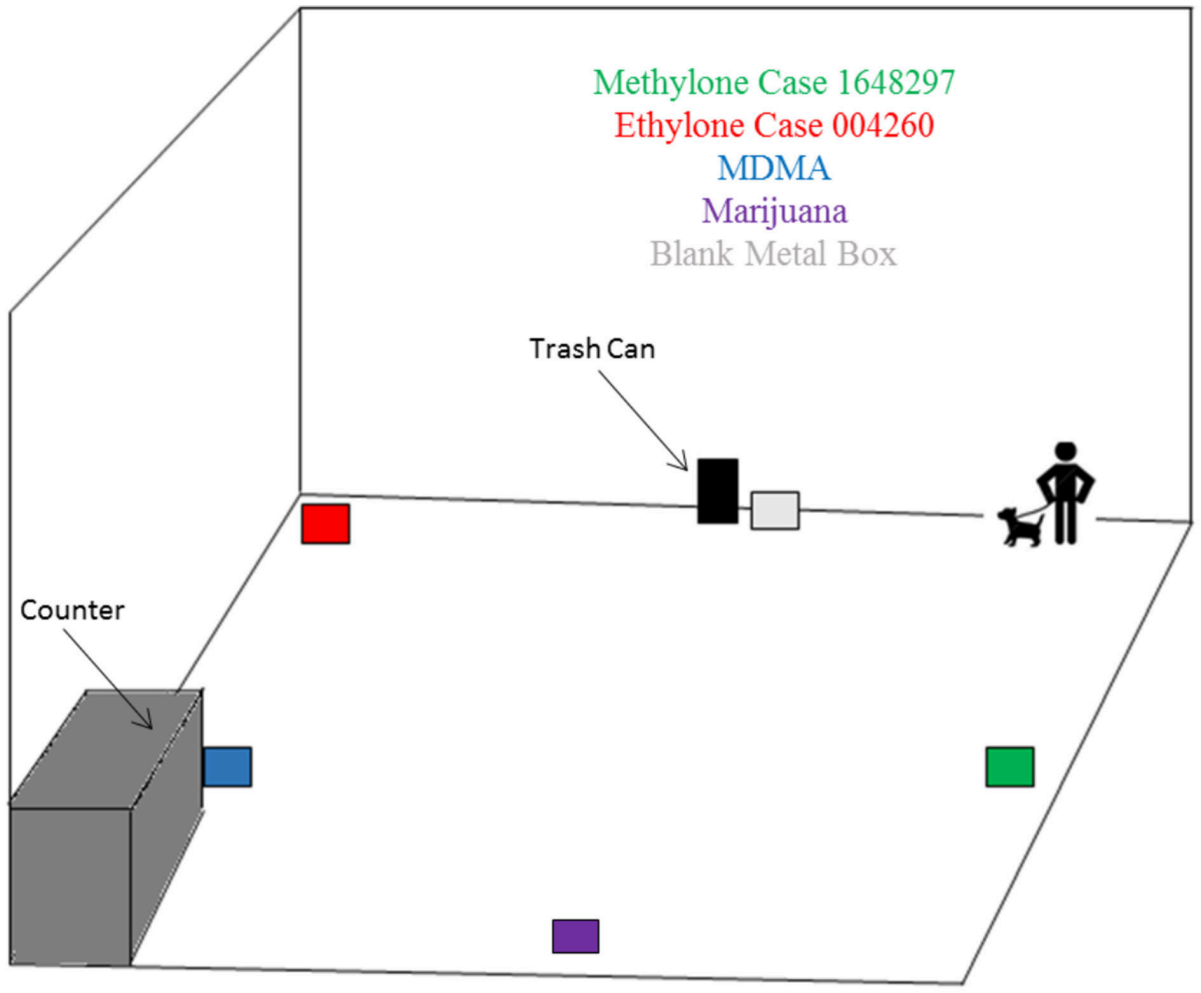
The schematic of the Miami-Dade Canine Trial (not to scale).

**Figure 2:**
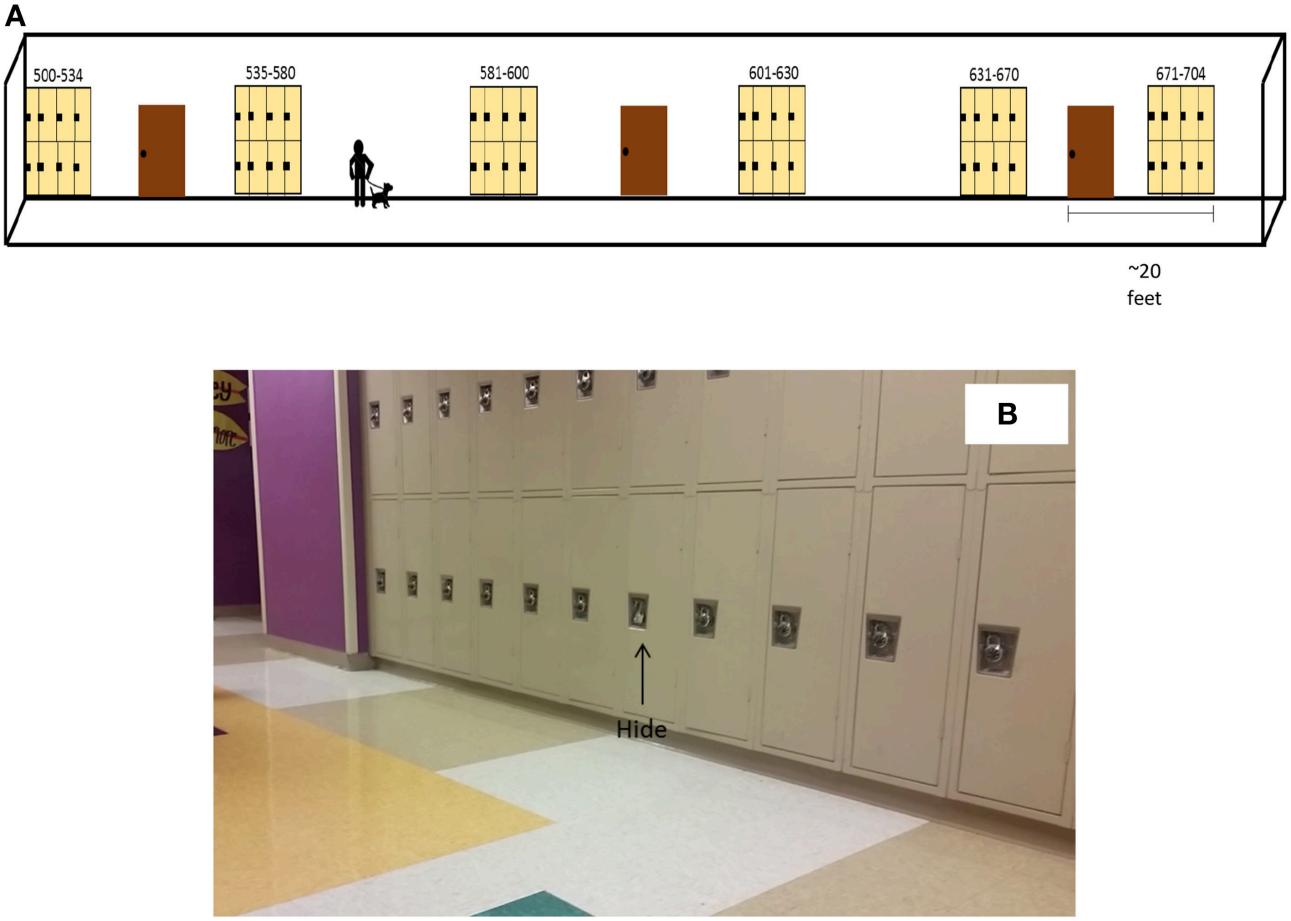
**(A)** Setup of Palm Beach Canine Trial **(B)** Picture of lockers.

Prior to imprinting, preliminary canine trials were conducted at both the Miami-Dade and Palm Beach facilities, as demonstrated in Figures 1, 2. These canine trials were used to assess whether the canines could detect synthetic cathinones while only being imprinted on the substances previously listed. For the Miami-Dade trial, the allotted space used was the lab's cafeteria, which provided a connecting outside ramp for easy access. The section used for testing was closed off and controlled. As depicted in the diagram the hides consisted of the methylone and ethylone for testing, the positive control, and a blank. The blank did not contain any odor which would cause an alert by the canine. The positive controls (confiscated marijuana and 3,4-methylenedioxymethamphetamine) and the cathinones were located at the least 10 feet apart. Each case and control was placed in pre-washed metal boxes provided by the canine teams; also used during routine training. Each hide was allotted a minimum of 30 min for the volatile organic compounds (VOCs) to be released and made available for the canines to detect.

Canine trials were conducted in a single blind test scenario as the evaluators knew the outcome but the canine-handler team did not. Each canine team was allowed to search the room in the same manner in which they would typically conduct a normal search. The handlers were instructed to inform the observer whether their dog alerted, showed interest, or failed to alert to the hides. The canine handlers were also informed that they could only reward their canines if they alerted to the positive control, as confirmed by the researcher, and were instructed not to reward their canines for alerting to a hide that contained any bath salt. As each canine team completed their first run, they were ushered out of the testing area, the order of the teams were again randomized and the teams performed a second and third run after being randomized again. The canine trial in Palm Beach County was set up in the manner depicted in Figure 2 (2 runs per team).

Each synthetic cathinone or hide was placed in a separate locker corridor. Only the bottom half of the lockers were used to optimize canine odor interaction as the trainer had previously conducted canine work in this manner. The cases were supplied by PBSO through the required standard for drug retrieval for canine detection work. Each case arrived in heat seal non-permeable bags and was opened to retrieve the inner bags. Each case contained approximately 10 grams of the bath salt. All designated lockers were opened and with gloved hands, the inner bags were taken out and placed in the seam of the locker. The lockers were then closed and the locks were fixed in a manner similar to the unopened surrounding lockers.

Similar instructions were given to the canine handlers at Palm Beach as were given in the Miami-Dade canine trials. The alert, interest, and failure to alert indicated by the handlers were noted.

After the preliminary trials, this study's imprinting phase was completed utilizing PBSO's canine detection team. These canines were divided into two sets, those imprinted on α-PVP only and those imprinted on ethylone. Cleaned mason jars containing the selected material were screwed into the open slot within a Behavior Shaping Device (K-9 BSD-2 kit; popper box). Clean hand towels (rewards) were rolled, taped in place and positioned in the shooter hole (open slots attached to the device's back) of the BSD. For imprinting purposes 5 boxes were used; one box contained the target odor and the remaining four boxes were blank. The odor was allowed to accumulate for 30 min before allowing canines to search. Canines were led to walk along the boxes and brought to the odorant box where it was allowed to sniff. At this point, the BSD would eject the towel (reward) from the box and the canine was allowed to grab the reward. This was repeated at least three times per session (3 sessions per week).

The second phase objective was to develop the searching pattern. The canine was brought in and given the command to search, if the canine passed the odorant box, they were corrected by the handler pulling on the leash and brought back to be reintroduced to the box. When the canine remained stationary at the box, the shooter ejected the towel and the canine collected its reward. This process was repeated for several weeks until the dog was able to sit or alert. The third phase incorporated distractors to test the canine's focus, alertness, sensitivity, and reproducibility of positive indications. The boxes remained idle for the first 30–40 min prior to the trial. The canines were given the active command to search. Corrections were given where needed, until satisfactory results were achieved for the trainer.

After completion of the imprinting process a validation test was conducted. The seized bath salt cases were placed in unmarked boxes approximately 10 feet apart. The search included distractors and MDMA as the positive control. Based on the guidelines following SWGDOG, the canine teams conducted the search and the alerts were recorded as indicated by the handler. The canines had to receive a minimum score of 90% correct responses to be confirmed as being successfully imprinted on the new drug. Canine teams that scored lower than 90% were asked to reinforce the imprinting process and perform the validation test after another week.

## Results

### Initial Response of Narcotic Detection Canines to Bath Salts

As synthetic cathinones are structurally similar to both MDMA and methamphetamine, it was initially theorized by law enforcement officials that their odor would be similar to bath salts. This led to the expectation that those similarities would allow canines that can detect MDMA and methamphetamine, to also successfully detect synthetic cathinones. However, as shown in Tables 1, 2, all of these certified narcotic detection canines (*n* = 12), though able to alert to the presence of their positive controls (PC1) and (PC2), failed to alert to bath salts.

**Table 1:** Detection capabilities of narcotic detection teams deployed in Miami-Dade County.

**Canine No**.	**PC 1 (Marijuana)**	**PC 2 (MDMA)**	**Methylone**	**Ethylone**	**Blank**
1	3/3	3/3	0/3	0/3	0/3
2	3/3	2/3	0/3	0/3	0/3
3	3/3	3/3	0/3	0/3	0/3
4	3/3	3/3	0/3	0/3	0/3
5	3/3	3/3	0/3	0/3	0/3
Alert Response	100%	94%	0%	0%	0%

**Table 2:** Detection capabilities of narcotic detection teams deployed in Palm Beach County.

**Canine No**.	**PC 1 (Marijuana)**	**PC 2 (MDMA)**	**αPVP Case # 17–426**	**αPVP Case# 14–1856**	**Ethylone Case # 15–02913**	**Ethylone Case # 14–65213**	**Blank (Currency)**
1	2/2	2/2	0/2	0/2	0/2	0/2	1/2
2	2/2	2/2	0/2	0/2	0/2	0/2	0/2
3	2/2	2/2	0/2	0/2	2/2	0/2	0/2
4	2/2	2/2	0/2	1/2	0/2	0/2	0/2
5	2/2	2/2	1/2	0/2	0/2	1/2	0/2
6	2/2	2/2	0/2	0/2	2/2	0/2	0/2
7	2/2	2/2	0/2	0/2	0/2	0/2	0/2
Alert Response	100%	100%	7.1%^*^	7.1%^*^	28.5%	7.1%^*^	7.1%^*^

* *Interest Rate Calculated when canine showed interest but no alert was confirmed by the handler*.

A more detailed evaluation of the Palm Beach trial (Table 2) revealed that canines could not reliably alert to synthetic cathinones such as α-PVP and ethylone. The interest percentage was approximately 7% (negative predictive value was 95%). Although there was interest shown for the three baths salts in Table 2, no canine produced a final alert. The confiscated currency used as a blank during this trial also produced some interest by one canine during the first run but no response upon the second exposure. The Ethylone had an overall alert rate of approximately 28% with a low PPV (positive predictive value) of 27%.

### Imprinting of Canines on Bath Salts

In order to correct the inability of canine teams to detect bath salts, they were divided and imprinted on two types of synthetic cathinones. This division was used to test the ability of each canine to accurately detect the presence of other cathinone derivatives, even though they may not have been previously imprinted on them. Introducing the canine to the odor was the first stage of imprinting. The popper boxes employed by the trainers were devices equipped with a launcher that housed a reward and ejected it for positive reinforcement. With this device, it was noticeable that the canines appeared to develop odor recognition rapidly in comparison to other methods of rewarding for imprinting (i.e., towel tugging and PVC pipes). During the odor introduction stage, after the first session all canines began to familiarize themselves with the odor associated with each specific bath salt. However, the odor introduction continued for 1 week.

During the search pattern stage of imprinting, improvement was observed for the canines. One canine team in particular developed a strong alert to α-PVP after the first week, indicated by the handler's attempt to solidify the canine's confidence by employing the “walk-away” method. This method is where the handler will actively walk away to test whether the canine will break from their alert or hold fast. Nonetheless, all canine teams were able to actively search and identify the presence of each bath salt after approximately two and a half weeks of routine imprinting sessions.

The last stage of the imprinting process incorporated distractors such as dog food, tennis balls, and play toys. During this phase four of the canine teams struggled initially. The canines were continuously given various commands from the trainer and handler and removed from the active search line as part of a corrective measure. Each corrective action was performed to reinforce the canine's drive to actively search and detect (work vs. play). This part of the training required the most work for these canine teams with successful completion after approximately 1 month of onsite and at home reinforcement. After completion of the imprinting process, 12 canines were tested for validation; two separate test days. Using the same samples from Table 2, the validation trial concluded that all canines had been successfully imprinted on the odor of these drugs. The canines imprinted on α-PVP (group A) were able to detect the Ethylone cases and the same was witnessed with group B (imprinted on the Ethylone); combined alert rate of 100% (based on 2 canine trials).

## Discussion

Assessment into the detection capabilities of currently certified narcotic detection canines reveals that they failed to reliably alert to synthetic cathinones (bath salts). Headspace analysis of confiscated bath salts, methylone, ethylone, α-PVP and 3,4-methylenedioxypropiophenone using Solid Phase Microextraction has revealed that these bath salts do in fact have different headspace profiles, these results have been previously reported (22). However, substantial overlap does exist with compounds such as methylone being detected in the headspace of all the confiscated samples allowing for canines to use one or more of these compounds as the active odorant that is responsible for them producing an alert. Studies are ongoing to further isolate these active odorants to ultimately create a mimic canine training aid for the detection of synthetic cathinones. More than 85% of the canines tested in this study from both counties (26 of the 29 runs) were not able to detect any of the bath salt cases presented; while only 20% (Palm beach canine teams; 3 of the 14 runs) showed interest in the bath salts without producing a final alert. Twelve canines were successfully imprinted on confiscated bath salts within a 1 month period. The canine trials conducted have shown that certified narcotic detection canines can in fact be quickly imprinted and trained to detect these new threats within a matter of weeks with sufficient reliability to pass a certification with 90% accuracy. Testing also revealed that canines that were imprinted on one type of bath salt α-PVP (group A) were able to detect the Ethylone and the same was witnessed with those imprinted only on Ethylone (group B) who were able to alert to α-PVP. Analytical headspace analysis using solid phase microextraction on several ethylone and α-PVP samples revealed compounds common in both samples, helping to explain how canines are able to detect either bath salt. Further studies are being conducted to identify and isolate the active odorant in these bath salts responsible for a canine alert, which can be used to create a safe and reliable mimic training aid for canine teams.

## Ethics Statement

This study was carried out in accordance with the recommendations of The Institutional Animal Care and Use Committee (IACUC) at FIU. The protocol was approved by the FIU Animal Welfare Assurance.

## Author Contributions

VS conducted all experiments described and data analysis within this manuscript. She was also the lead author responsible for recording and assembling the text within this manuscript. HH assisted in the experimental design, data analysis, and writing of the manuscript described. KF designed, funded and extended collaborative partnerships used in the execution of this project. He was also responsible for reviewing and crafted conclusions of the manuscript.

### Conflict of Interest Statement

The authors declare that the research was conducted in the absence of any commercial or financial relationships that could be construed as a potential conflict of interest.
